# Living with Multimorbidity through Time: A Meta-Synthesis of Qualitative Longitudinal Evidence

**DOI:** 10.3390/healthcare12040446

**Published:** 2024-02-09

**Authors:** Cheng Cheng, Martin Christensen

**Affiliations:** 1 School of Nursing, The Hong Kong Polytechnic University, Hung Hom, Kowloon, Hong Kong SAR, China; cheng123.cheng@polyu.edu.hk; 2 The Interdisciplinary Centre for Qualitative Research, School of Nursing, The Hong Kong Polytechnic University, Hung Hom, Kowloon, Hong Kong SAR, China; 3 School of Nursing, Fudan University, Xuhui District, Shanghai 200032, China

**Keywords:** chronic disease, longitudinal studies, review, qualitative research, long-term care, multimorbidity

## Abstract

The growing prevalence of multimorbidity places a strain on primary healthcare globally. The current study’s aim was to identify, appraise, and synthesize published qualitative longitudinal research on individuals’ experiences concerning living with multimorbidity through time. The authors searched two electronic databases, MEDLINE and CINAHL, and performed an additional literature search in Google Scholar. A thematic synthesis approach was used to analyze the qualitative data across the studies. A total of 10 reports that met the inclusion and exclusion criteria were included in the synthesis. Five descriptive themes emerged from the analysis of the living experiences of individuals with multimorbidity: (1) perceiving multimorbidity, (2) managing chronic conditions, (3) emotional struggles in everyday life with multimorbidity, (4) interactions with the healthcare system and healthcare professionals, and (5) family support. This meta-synthesis provides insights into the diverse perceptions of multimorbidity and how individuals cope with their chronic conditions in their daily lives. The findings highlight the importance of establishing effective patient-centered care that acknowledges and supports the multifaceted needs of this population. It is also recommended to involve a psychological component in the care of individuals with multimorbidity, as part of a collaborative and interprofessional approach.

## 1. Introduction

Multimorbidity, the coexistence of two or more chronic conditions in an individual, is a growing concern in healthcare [1]. As population age increases, the prevalence of multimorbidity continues to rise globally [2] and poses substantial challenges for individuals [3], healthcare professionals [4], and healthcare systems alike [5].

While quantitative research has documented strong evidence of the prevalence and impacts of multimorbidity, qualitative studies offer a unique perspective on the experiences and challenges faced by individuals living with multimorbidity. These studies provide an in-depth understanding of how individuals navigate their lives, manage their health, and interact with healthcare systems over an extended period [6,7,8].

Multimorbidity impacts healthcare processes and may result in compound care needs [9]. There is, at present, a need for interventions to improve healthcare in this population. A better understanding of individual transitions among multimorbidity over time may help researchers to develop tailored interventions and improve patient outcomes.

A preliminary search has identified several qualitative reviews in the context of multimorbidity: Rosbach and Andersen [10] conducted a meta-ethnography to identify the component of treatment burden in multimorbid patients and investigate their strategies of management. Shin et al. [11] reported that older adults with multimorbidity may struggle with manifold barriers and challenges caused by functional deterioration and social isolation in everyday life. Another two reviews presented how healthcare professionals such as physicians and nurses understand multimorbidity’s impacts and deliver effective healthcare for this population [6,12]. The experiences of multimorbidity are, thus, well described; however, no synthesis of longitudinal qualitative research on individual experiences with multimorbidity is available to offer evidence for policymakers, healthcare practitioners, and further research.

Qualitative longitudinal research (QLR) refers to qualitative studies that involve repeated data collection over time, with an emphasis on the temporal aspects of a particular phenomenon such as living with chronic diseases. There has been a growing interest in the use of QLR in healthcare research, as it is particularly appropriate for exploring topics which involve changes over time, such as the progression of chronic diseases [13]. The authors of this paper aimed to address this knowledge gap by undertaking a review of this topic. This meta-synthesis will contribute to the existing body of knowledge on multimorbidity by providing a comprehensive overview of the lived experiences of individuals over time and help pave the way for future practice to improve multimorbidity care.

This study’s aim was to identify, appraise, and synthesize published qualitative longitudinal studies that describe how individuals live with multimorbidity through time. The primary research question of this study was the following: what are the individual experiences of living with multimorbidity through time?

## 2. Materials and Methods

### 2.1. Research Design

Qualitative meta-synthesis is a systematic and cohesive way to analyze data from multiple qualitative studies. It is a technique that allows researchers to identify a specific research question and is increasingly considered a powerful tool for evaluating participants’ meanings, experiences, and viewpoints [14]. The authors of this paper adhered to a six-step qualitative meta-synthesis approach [15]: (1) identifying a clear research question, (2) conducting a comprehensive literature search, (3) performing a quality evaluation with a reliable instrument, (4) extracting important data, (5) analyzing data with a well-known method, and (6) presenting the findings. Figure S1 depicts the whole procedure of meta-synthesis of this study.

The authors used the ENTREQ (enhancing transparency in reporting the synthesis of qualitative research) statement to ensure rigor in reporting this synthesis [16].

### 2.2. Literature Search

The authors conducted a conventional pre-planned search in MEDLINE and CINAHL using a searching framework on the 5 October 2023 and updated it on the 10 December 2023. The authors applied the MeSH terms and the free terms in the search. Table S1 reports the details of the literature search for this study.

This meta-synthesis was not registered while a preliminary search was being performed in MEDLINE, CINAHL, and COCHRANE databases to ensure no similar study had been completed beforehand. A supplementary search in Google Scholar was performed. Moreover, the reference lists of the most relevant qualitative literature reviews were also examined [6,11,12,17]. No timeframe of publication was applied in the search approach as the authors sought to gain an overview of the existing evidence.

### 2.3. Eligibility Criteria and Study Selection

The search results were brought into EndNote^TM^ 21 for screening purposes. Once duplicate entries were removed, a two-step screening process was applied, which involved screening titles and abstracts as well as conducting a full-text check. The authors specifically included longitudinal qualitative studies that examined the experiences of individuals with multimorbidity. The primary author assessed all potential reports based on predetermined criteria for inclusion and exclusion. An experienced qualitative researcher reviewed and supervised the screening process. In case of any disagreements, these were resolved through discussion. Table S2 shows the inclusion and exclusion criteria of this study.

### 2.4. Quality Appraisal

The authors used the Critical Appraisal Skills Programme (CASP) qualitative studies’ checklist to evaluate the quality of the included studies [18]. The CASP tool is suggested as a recognized and widely-used tool for quality assessment in healthcare qualitative evidence syntheses [19]. The primary author evaluated all potential reports based on the CASP checklist, and an experienced qualitative researcher administered the appraisal process. Any disagreements were resolved through the census.

### 2.5. Data Extraction

The authors extracted the key information from each included study with a designed table. The extracted information included the following: author (publication year) and country, aim, design, setting and sample, data collection process, and data analysis. The primary author inserted the above information into the table, and then an experienced researcher reviewed it. All disagreements regarding the extracted information were resolved by discussion.

### 2.6. Data Analysis

The authors examined the original data from each study using an inductive thematic synthesis approach, which is critical given the study’s aim to generate high-order themes based on the living experiences of people with multimorbidity [20]. A three-step analytical process was applied as follows. (1) Unrestricted line-by-line coding of primary study results to identify “free codes”: In this stage, the authors initially extracted and synthesized findings according to the aims of this synthesis regarding lived experiences with multimorbidity. The authors entered the findings of these studies into an electronic online database, then coded each line. (2) Grouping these “free codes” into related areas to create “descriptive” themes (sub-themes): In this stage, the authors identified similarities and differences between the codes and began to categorize them into a tree structure. (3) Developing “analytical” themes (major themes): In this stage, the authors developed “third-order interpretations” which abstracted the content of the original studies. The authors repeated this process until no new themes were found. The authors discussed the combined findings at each stage. The analytical process of this study was aided by the Taguette [21].

## 3. Results

### 3.1. Search Results

**Figure 1:**
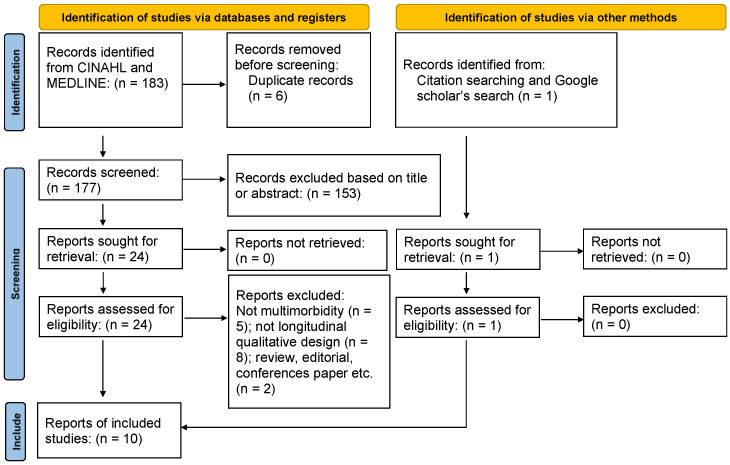
Procedure of selection.

Figure 1 illustrates the procedure of searching and screening using the Preferred Reporting Items for Systematic Reviews and Meta-analyses (PRISMA) flow diagram [22]. Of the 183 records yielded, 6 were duplicates; 153 were excluded based on their title or abstract; and 24 reports were reviewed in full. A total of 10 reports were included finally. The full-text review was performed by the primary author, and nine reports were retained. An additional report was identified from the supplementary search.

### 3.2. Quality Appraisal

Table S3 shows the quality appraisal of the included reports using the CASP checklist. The answer options for the questions were yes (Y), no (N), and unclear (U). Twelve items were scored as 0 (=unclear or no) and 1 (=yes). Based on the points scored, a study was placed in one of three possible groups: high quality (9–12), moderate quality (6–8), and low quality (0–5). All the included studies were marked as “high quality”, and no study was excluded based on the quality appraisal.

### 3.3. Overview of the Included Studies

Table 1 shows a summary of key information from the included reports. The 10 reports were spread throughout five Western countries including the USA (n = 1) [23], New Zealand (n = 2) [24,25], Sweden (n = 1) [26], Canada (n = 1) [27], and the United Kingdom (n = 5) [28,29,30,31,32]. Together, 335 participants in various settings, reporting experiences regarding multimorbidity, were included in the review. The majority of participants were older adults (aged over 60 years old). Purposive sampling was used mostly to recruit participants (n = 6). Face-to-face semi-structured interviews were used in all the studies to collect data, and several supplementary methods such as field notes were also employed. The data analysis techniques were varied: thematic analysis combined with other approaches (n = 4), inductive approach (n = 2), narrative approach (n = 1), deductive and inductive coding (n = 1), grounded theory (n = 1), and iterative framework (n = 1).

### 3.4. Findings of the Thematic Analysis

Five main themes emerged from our synthesis. Participants discussed the physical and psychological toll that living with a chronic illness takes on their lives as well as how they viewed their conditions and the strategies they had taken to manage them. The participants described their needs during the medical encounters and berated the lack of clear communication from healthcare providers, which might leave them feeling unsupported. The value of family support was also talked about by the participants. Figure S2 depicts the coding tree for the thematic analysis of this study.

#### 3.4.1. Perceiving Multimorbidity

The theme of “perceiving multimorbidity” revolves around personal experiences and perceptions of health, illness, and aging. The participants discussed their various health conditions, including diabetes, epilepsy, high blood pressure, arthritis, and cancer, and how these conditions affected their daily lives. They also touched on the physical changes and inconveniences brought about by aging, such as difficulty sleeping, breathlessness, and physical slowdown.

**Identity and illness.** The individuals’ identities were significantly influenced by their health conditions, with one person identifying themselves as “*a patient*” rather than “*a normal person*”. This suggests that their illnesses had become a defining part of their identities.

**Impact of chronic illness.** The participants repeatedly highlighted the debilitating effects of chronic illnesses like COPD, arthritis, fibromyalgia, and potential Alzheimer’s. These conditions severely limit the individuals’ physical capabilities, affecting their mobility, breathing, memory, and quality of life. For example, a participant stated the impacts as follows: “*But this breathing, this COPD that’s what will affect me in another few weeks when it starts getting cold; it’s the cold that affects me*”.

**Prioritization of health problems.** Due to the various clusters and trajectories of multimorbidity, the participants usually determined an order of prioritization between their different chronic conditions, which was largely driven by their diseases and symptom experiences. For example, a participant described the following prioritization: “*Probably blood pressure, probably and cholesterol… so I’m more worried about those because they are more serious things. IBS didn’t kill anybody, you know, but blood pressure is serious and cholesterol is serious so IBS has gone into the background, you know*”.

**Medication burden.** Medication is a dominant method of controlling chronic conditions. The participants reported frustration and confusion regarding the number of medications they had to take, the side effects, and the difficulty in keeping track of them all. For example, a participant said the following: “*I started off with one medication, then I was on two, then three, then the heart*”.

**Aging and health.** From the views of the participants, aging was seen as a contributing factor to their chronic conditions, with the individuals acknowledging that their age made them less agile and more vulnerable to multimorbidity. For example, a participant noted their opinions regarding age as follows: “*Breathing. Um, only that I can’t do the things I used to, but whether that’s breathing or whether that’s old age I’m not sure*”.

#### 3.4.2. Managing Chronic Conditions

Despite their multiple health issues, the participants aimed to maintain a positive outlook, choosing to view these conditions as inconveniences rather than illnesses. They showed a desire to stay healthy and ways of keeping a good quality of life. They also emphasized the position of mental health, stating that their brain was still alright despite their body “falling apart”.

**Adaptation and resilience to illness.** The participants were seen adapting to their health conditions using various strategies, such as taking taxis instead of walking, planning activities around their energy levels, and using medication and regular check-ups to manage their conditions. They also mentioned the importance of diet in managing their conditions. This shows their proactive approach towards their health despite their limitations. Despite the challenges, they showed acceptance of their conditions as part of aging and demonstrated resilience in managing their health. For example, a participant listed the following strategies of management: “*I have to sort of eat regularly for epilepsy, eat regularly, not get over tired, not drink too much alcohol… don’t skip meals, so that is what I do for me epilepsy anyway, so that’s what helps with diabetes as well*”. In addition, a participant also valued the importance of management: “*Well she talked about diet and um, yes really it was diet really you know, just be careful what I eat*”.

**Self-sufficiency and autonomy.** The participants expressed a strong willingness to be self-sufficient and not a burden to others. They also expressed a desire for autonomy and control over their health, wanting to ensure that their treatment was correct and that they would be involved in decision making. For instance, a participant noted the following: “*I want to be self-sufficient. I don’t want to be sick. Until I’m overwhelmed, I want to be able to deal with it [my illness] on my terms*”.

**Goal setting and determination.** The participants discussed the difficulties of setting and achieving health goals, particularly around weight loss. They stated feelings of failure when they could not meet their goals and questioned the usefulness of goal setting in their situation. They also showed a determination to keep their conditions under control and to continue living their life as fully as possible. For example, a participant showed determination in coping with multimorbidity: “*I want to fight it with all I can get. No short cuts*”.

#### 3.4.3. Emotional Struggles in Everyday Life with Multimorbidity

For the theme regarding emotions and multimorbidity, the participants depicted the impact of their conditions on their mental health, leading to emotional struggles, such as feelings of despair and frustration and the feeling of merely existing rather than living, etc. They felt overwhelmed by the constant changes and challenges that came with managing their health, and they struggled to keep up or find the energy to continue.

**Despair and hopelessness.** The participants frequently expressed feelings of despair and hopelessness and a lack of motivation. They felt that their situation was beyond repair and that their efforts to improve it were useless. This is evident in statements such as “*This can’t be fixed, the damage has been done*” and “*I’ve nothing to live for anyway, diabetes will kill me anyway*”.

**Frustration and stress.** The participants felt frustrated and stressed by their health condition and the associated challenges. They expressed frustration with their inability to make progress towards their goals and stressed over the prospect of further medical procedures. For example, a participant showed the following: “*long-term goals… keep getting pushed back—I’ve probably had the same ones for years. Lose weight and get fit. And I have done the opposite. I tend to leave my goals in the car park [at the health centre] when I leave… I get so frustrated by my lack of progress*”.

**Grief and loss.** The participants spoke of an ongoing sense of grief and loss, referring to it as “*a lifetime of letting go*”. They felt that they had lost everything and were constantly having to adjust to new challenges and losses.

**Self-blame.** The participants blamed themselves for their situation, as seen in the statement “*I had a fall… my fault doing something stupid*”. This suggests feelings of guilt and self-blame, which could contribute to their feelings of despair and hopelessness.

**Lack of energy and motivation.** The participants frequently mentioned a lack of energy and motivation, stating “*I just haven’t got the energy to fight it*” and “*It’s like I’ve lost that thing—my mojo is it?—to do anything*”. This indicated a struggle with mental health, possibly depression, which was impacting their ability to cope with their physical health challenges.

**Fear and uncertainty.** There is a clear sub-theme of “*fear and uncertainty*”, particularly related to the progression of their illnesses and potential new health problems. This is seen in the worries about a worsening memory and the fear of losing sight. For example, a participant stated that “*This can’t be fixed, the damage has been done… I’m trying, I can’t. And it’s only these past months, to be quite honest with you, that I’ve had this, I’ve got to have this attitude, but I find it, I just don’t*”.

#### 3.4.4. Interactions with the Healthcare System and Healthcare Professionals

The theme of “interactions with the healthcare system and healthcare professionals” identified by the participants provided an impression regarding their encounters with healthcare staff, medical visits, and health systems. Their experiences illuminated specific areas of interactions that could be the emphasis of personal-level and system-level changes to improve the quality of care.

**Trust and honesty.** The participants hoped for their healthcare providers to be honest and transparent with them. They wanted to be told the truth about their conditions and the treatments they were receiving. They also wanted their healthcare professionals to be trustworthy and reliable. The participants expressed gratitude for their healthcare professionals and trusted them to make the right decisions regarding their treatment. Trust in healthcare professionals, including doctors and pharmacists, is also a key sub-theme, with the participants relying on their expertise to guide their treatment and manage their medications. For example, a participant stated the following: “*A person of trust that could tell me the truth… for example, if there was a solution that they would tell me ‘Francisco’ it’s all right. Having that confidence to give us that encouragement, right? That they tell me, you know what is good… that they do not put in doubt, but if they do make you doubt that they say ‘think about it.’ I would still appreciate it, right?*”.

**Personalized care.** The participants wanted their healthcare providers to understand their individual needs and circumstances. They wanted their healthcare professionals to put themselves in their shoes and provide treatment options that were best suited to their specific situations. They believed that this could allow for better communication and understanding of their treatment plan. For example, a participant noted the following: “*He said I don’t know why they’ve send you [Frances], I’ve nothing… he said what’s the matter? I said well, I don’t know, I said because I have everything what you’ve given me, so that were it*”.

**Confidence in treatment.** The participants wanted to feel confident in the treatments they were receiving. They wanted their healthcare professionals to prescribe the right medications and avoid unnecessary tests and procedures. They also appreciated the professionals’ ability to instill confidence in them. For example, a participant informed us of the following: “*They just kept saying it was arthritis but I was so weary. Then I went to see another doctor… and he said straight away what was the problem, and actually knowing you’ve got a problem it takes a lot of the stress away when people say they don’t believe you… once you know that you’ve got something you face up to it and you can tackle it better*”.

**Continuity of care.** The participants expressed frustration with having to see different healthcare providers who may not be familiar with their medical history. They wanted to have a consistent healthcare provider who knew them and their health conditions well. For example, a participant stated “*I have been assigned to him, I have not chosen a doctor myself and everything feels very uncertain. You have to start all over again and that is really hard*”.

**Empathy and understanding.** The participants wanted their healthcare professionals to show empathy and understanding. They wanted their healthcare professionals to be caring and supportive and provide emotional support when needed. For example, a participant noted the following: “*I couldn’t understand why she’d crossed it out. I mean she shouldn’t have done that… Four puffs four times a day is what it said on the prescription and I said add it up and when she added it up she realised, yes, I did need what I was getting but it took a lot of convincing with her. I was very angry over that*”.

**Health communication.** The participants valued open communication with their healthcare professionals, particularly their GPs and pharmacists. They actively sought clarification on their medication and treatment plan. The participants wanted their doctors to explain their conditions and treatments in a way that they could understand. They also wished for their doctors to listen to their concerns and answer their questions. For example, a participant said that “*Nobody explained what had happened at all in the hospital. All they were doing was making your chest better, which is fine and fair enough, but nobody ever said why I had got a bad chest*”.

The participants expressed frustration with the lack of clear communication from healthcare professionals, particularly around diagnosis and medication instructions. They felt that their doctors did not communicate effectively with them, leaving them confused and unsure about their condition and treatment. This is evident in the following statement: “*If you have undergone surgery or something at the hospital, you receive this note and the doctor rambles on a lot but then, when you get home, you wonder what they said*”.

**Healthcare system.** The participants discussed their interactions with the healthcare system, including surgeries, consultations, and diagnoses. They claimed some dissatisfaction with the medical care they had received, particularly concerning unresolved health issues such as urinary leakage after surgery. For example, a participant stated the following: “*As it is now, I have to chase every healthcare professional myself. It isn’t the role you should have when you are on sick leave. The whole idea should be that the system takes care of you, not that you should chase the system*”.

#### 3.4.5. Family Support

The theme of “family support” underscores the importance of family in supporting an individual’s health, particularly in the context of multimorbidity. The participants expressed gratitude for the support they received from their loved ones and emphasized the importance of open communication and involvement in their health decisions. The participants emphasized the role of their family in practical aspects of their care, such as collecting medication. Their speeches also touched on themes of planning for the future and considering end-of-life decisions.

**Family involvement and support.** The participants appreciated the involvement and support of their families in their health journey. They were open about their health status with their family and relied on them for decision making when necessary. For example, a participant reported that his daughter played a significant role in managing their medication. And, for instance, another participant noted the help of family members: “*I appreciate them, and all they’ve done for me. It builds confidence in people to talk to them, [to] keep stepping up and being there for you*”.

**Economic stability.** The participants expressed a desire to leave their loved ones in a stable financial situation. This could indicate concerns about the financial burden of their illness. For example, a participant said that they wished to “*leave the ones that I love economically stable*”.

**Advance care planning.** The participants acknowledged the need for advanced care planning, such as a living will or an advance directive, but had not yet discussed this with their families. For example, a participant said the following: “*This is something I would like to think about. I haven’t discussed this with my wife or family. I don’t have a living will or advance directive*”.

## 4. Discussion

This was a meta-synthesis of individuals’ experiences with multimorbidity over time. The emerging themes from the reviewed reports centered around the challenges of living with multimorbidity and its impact on people’s lives. Individuals with multimorbidity discussed the difficulties in maintaining a healthy lifestyle, expressed dissatisfaction with the healthcare system, and voiced a need for more information and support from healthcare professionals and family members. These themes also touched on aging, illness perception, and the negative emotions associated with chronic conditions.

In line with previous qualitative findings [33,34], this study confirmed that the resulting impacts of multimorbidity, such as functional impairment, disease-specific symptoms, and medication-related burden, significantly affect people’s daily lives. These impacts on multiple domains, including the physical, psychological, and financial ones and adherence to self-management, were also evidenced by several large cohort studies across continents [35,36,37,38], highlighting the diverse healthcare needs of multimorbid patients. Additionally, this synthesis identified the prioritization of chronic conditions, a finding that reinforced both quantitative and qualitative results [39,40], where people with multimorbidity prioritized a leading condition over other concurrent conditions. A systematic review showed a discrepancy between the priorities of multimorbid patients and healthcare professionals, with patients’ prioritization dominated by illness representation and healthcare professionals’ prioritization focused on long-term risks [41]. Further research on prioritization and potential pathways for reaching an agreement could help healthcare professionals to accurately identify their patients’ priorities and provide effective care.

Individuals with multimorbidity have to make daily adjustments based on their health status to manage their chronic conditions [42,43]. This synthesis revealed that most participants chose to maintain a positive attitude and focus on the diseases they could control, using strategies which promoted ownership of their health status. Several clinical trials have indicated that a positive attitude can have beneficial effects on one’s ability to manage daily activities in the context of chronic conditions [44,45,46]. Also, self-care in line with pre-set goals creates a sense of control over one’s health situation [47]. In line with other research findings [48], the participants in our reviewed studies tried to view their condition as “normal” and maintain some independence (autonomy). Normalization in the context of a chronic condition was seen as a way for individuals to resume their pre-illness roles and responsibilities, such as finding ways to engage in life [32,49]. Normalization is suggested as an effective strategy for maintaining psychological well-being and making people feel more comfortable with seeking help when they have to cope with other chronic conditions [50,51,52].

The relational theme concerning interactions between healthcare professionals and people with multimorbidity aligns with the principles of person-centered care [53] and has previously been discussed and interpreted by Kuipers et al. [54,55] and Poitras et al. [56]. The findings of this study, entirely based on patients’ experiences, revisited the key elements of Stewart’s definition, which stated that patient-centered care should include an exploration of the patient’s concerns and priorities for care, a sense of partnership between the patient and healthcare professional, and active patient involvement in decision making [57]. The findings of this study reflected major quality metrics for healthcare providers’ responsibilities, such as offering continuous care, sharing ongoing information with patients, considering feedback from patients, and coordinating with other healthcare professionals. These also aligned with the main elements of shifting from disease-specific care to patient-centered care, as proposed by the American Geriatrics Society [58]. Moreover, most participants reported unsatisfactory medical encounters due to care fragmentation, a common issue for people requiring long-term care [59]. Fragmentation could be an independent risk factor for adverse health outcomes [60]. Evidence from a cohort study showed that increased care fragmentation could lead to inappropriate medication and higher mortality rates [61]. Although patient-centered care is recommended for quality care in people with multimorbidity, not all aspects of patient-centered care are equally important to all due to heterogeneity in health-related domains such as physical function [55]. Therefore, the findings of this study highlighted the structural components required to realize patient-centered attributes. When translating those into practice, healthcare professionals in primary care settings should tailor their care to the needs of patients with multimorbidity to ensure the best possible outcomes. The optimal methods for achieving this type of patient-centered care in people with multimorbidity warrant further investigation.

Regarding how people with multimorbidity fared in terms of mental health, a previous meta-analysis found that people with multimorbidity reported more psychological distress compared to their peers without multimorbidity [62]. The theme of emotional struggles is one aspect that characterizes experiences related to multimorbidity, whether it involves feelings of despair, frustration, or hopelessness in dealing with physical limitations and treatments. These unpleasant feelings aligned with what other investigators have observed in people with different chronic diseases [63,64,65]. As multimorbidity progresses, these feelings might increase the risk of mental disorders such as depression, which complicates the treatment of existing chronic conditions [66]. The findings of this study underscored the importance of not neglecting the mental aspect of multimorbid patients.

### 4.1. Limitations

This meta-synthesis had limitations. First, this meta-synthesis is a summary of subjective dialogues, meaning that the findings which emerged might be affected by the author’s background and expertise. Next, the authors conducted a conventional search with terms regarding multimorbidity. This likely resulted in related studies from the perspectives of patients with chronic conditions not being included in the analysis. Also, the restriction to studies published in English might have resulted in the exclusion of relevant studies conducted in other languages. Another weakness was the fact that the primary author played a key role in obtaining and processing the data independently. However, during data analysis, these were reviewed afterward by the second author. Last, this study used the CASP qualitative studies’ checklist to evaluate the reports included in this synthesis as there was no specific reporting guideline for longitudinal qualitative research.

### 4.2. Implications

The findings of this synthesis had implications for practice. First, how individuals perceive their multimorbidity guides how they cope with their conditions and management. Inaccurate perceptions may contribute to treatment gaps and underestimation of health risks in the context of chronic conditions. An enhanced understanding of illness representations in multimorbidity may provide a useful component for making sense of relationships and prioritizing competing demands from several conditions. Next, this study reflects several essentials of establishing person-centered care for people with multimorbidity. Despite the fact that there might be some ambiguity in treatment priority between patients and healthcare professionals as well as the fact that care fragmentation remains an existential issue in the provision of integrated care, sufficient and accurate resources, constant attention to the patient–professional relationship, and facilitation of communication may contribute to better healthcare efforts. Finally, since the strong association between multimorbidity and psychological distress has been proved, mental health needs to be addressed as a matter of urgency and on several levels, from optimizing the healthcare system to building collaborative care models. For example, this could be achieved by providing precise assessment and regular monitoring for high-risk groups, offering necessary training for healthcare professionals, and integrating psychiatric services with healthcare plans in primary care settings.

For policymakers, the findings of this study highlight the need for healthcare systems that are equipped to manage multimorbidity. This contains healthcare policies that promote integrated and coordinated care as well as adequate resources for services that address the complex needs of patients with multiple chronic conditions. Also, this study suggests that current healthcare policies may not adequately address the needs of patients with multimorbidity. Policymakers should consider improving these guidelines to better accommodate the wants of people with multimorbidity.

## 5. Conclusions

In essence, the current synthesis offers an intimate look into the experiences of living with multimorbidity and the associated challenges in terms of impacts and management. This synthesis underscores the significance of maintaining a positive outlook, adjusting to physical changes, and advocating for one’s health. The findings of this study fill a crucial void in multimorbid-related studies by pinpointing key elements of patient-centered care across various diagnoses, countries, and healthcare settings, highlighting the necessity for healthcare professionals to expand their viewpoints through individuals’ lived experiences. Furthermore, this study provides a touching examination of the emotional struggles endured by individuals living with chronic diseases, emphasizing the need for improved mental health support and more transparent communication in healthcare environments.

## Figures and Tables

**Table 1:** Key information of the included reports.

Author (Year) & Country	Aim	Design	Sample	Data Collection Process	Data Analysis
Morris et al. (2011), UK [28]	To explore factors that impact self-management priorities for people with multimorbidity and their changes.	Longitudinal qualitative study	Purposive sampling. 21 participants (Female: 11) with multimorbidity. Ages 36–84 years. Common diseases were diabetes and COPD.	Face-to-face interviews, telephone follow-ups, and final face-to-face interviews a year later	Semi-structured and narrative approach
Mason et al. (2016), UK [29]	To explore the experiences and perceptions of people with multimorbidity.	Serial multi-perspective interviews	Purposive sampling. 37 participants (Female: 14), mean age 76 years, range 55–92 years. Common diseases were heart, respiratory, liver, and renal failure, lung cancer, neurological conditions, and mild dementia.	Semi-structured serial interviews with people with multimorbidity and their family carers at 8–12 weekly intervals	Thematic analysis, cross-case analysis, and interpretive analysis
Naik et al. (2016), USA [23]	To explore health-related values concerning care in older adults with multimorbidity.	Qualitative component of a longitudinal mixed-methods study	146 participants (Female: 3), 107 aged >60 years. (73.3%). Common diseases were diabetes mellitus, chronic pulmonary disease, peripheral vascular disease, and cerebrovascular disease.	Open-ended questions and interviews with 6-month intervals	Deductive (a priori) and inductive (emerging from the data) coding approaches
Hays et al. (2017), UK [30]	To explore threats to patient safety in older adults with multimorbidity.	Longitudinal qualitative study	Purposive sampling. 26 participants (Female: 15), mean age 76 years, range 66–87 years. Common diseases were painful and respiratory conditions, hypertension and coronary heart disease, thyroid and prostate disorders, diverticular and chronic kidney disease, diabetes, anxiety, stroke, psoriasis, and glaucoma.	In-depth semi-structured interviews	Thematic analysis with a framework approach
Daker-White et al. (2018), UK [31]	To explore safety issues in people with multimorbidity.	Ethnography and longitudinal qualitative study	Purposive sampling. 25 participants (Female: 14), all aged over 65 years.	Face-to-face interviews every 12 months observation, and field note material were also collected	Thematic analysis
Francis et al. (2020), New Zealand [24]	To explore experiences of long-term care in people with multimorbidity.	Qualitative, multiple cases	16 participants (Female: 9), most aged 50–69 years (68.8%). Common diseases were diabetes, heart diseases, COPD, and hypertension.	Four weekly face-to-face semi-structured interviews	Narrative inquiry and thematic analysis
Porter et al. (2020), UK [32]	To explore lived experiences of multimorbidity from the point of view of patients.	Longitudinal qualitative study	Purposive sampling. 15 participants (Female: 8), all aged 65+ years. Common diseases were osteoarthritis and cardiovascular disease including hypertension, heart disease, and heart failure.	Two in-depth qualitative interviews from three to six months	Constructivist grounded theory
Brandberg et al. (2021), Sweden [26]	To explore self-management challenges in people with multimorbidity.	Longitudinal qualitative study	Purposive sampling. 16 participants (Female: 7), mean age 71 ± 10 years. Common diseases were congestive heart failure, COPD, hypertension, diabetes, renal failure, and anemia.	Four to five interview sessions per patient, seventy recorded sessions in total	Inductive qualitative content analysis and longitudinal analysis
Bravo et al. (2022), Canada [27]	To explore the experiences of patient–provider relationships among older foreign-born Latinos with multimorbidity.	Longitudinal qualitative study	Convenience sampling. 13 participants (Female: 10), mean age 75 years, range 65–85 years. Common diseases were unknown.	Three rounds of semi-structured in-depth qualitative interviews over nine months	Inductive approach
Collier et al. (2023), New Zealand [25]	To explore the experiences of older people with frailty, multimorbidity, and polypharmacy regarding the role of pharmacists.	Longitudinal ethnographic study	20 participants, age range 68–89 years. Eight participants (Female: 6) followed up	Semi-structured interviews, observation field notes, and photographs	Iterative framework

## Data Availability

The data supporting this study’s findings are available from the primary author, upon reasonable request.

## Supplementary Materials

The following supporting information can be downloaded at: https://www.mdpi.com/article/10.3390/healthcare12040446/s1: Table S1: Reporting of database searching; Table S2: Inclusion and exclusion criteria; Table S3: Methodological quality assessment [23,24,25,26,27,28,29,30,31,32]; Figure S1: Meta-synthesis procedure; Figure S2: Coding tree for thematic analysis.
