# Supplementary material for: Living with Multimorbidity through Time: A Meta-Synthesis of Qualitative Longitudinal Evidence
Source: Healthcare (Basel). 2024 Feb 9;12(4):446. doi: 10.3390/healthcare12040446 (PMC10887575; doi:10.3390/healthcare12040446)
Supplement: Supplementary file 1 [file healthcare-12-00446-s001.zip › Supplementary Figures.pdf]

PRISMA 2020 flow diagram for new systematic reviews which included searches of databases, registers and other sources

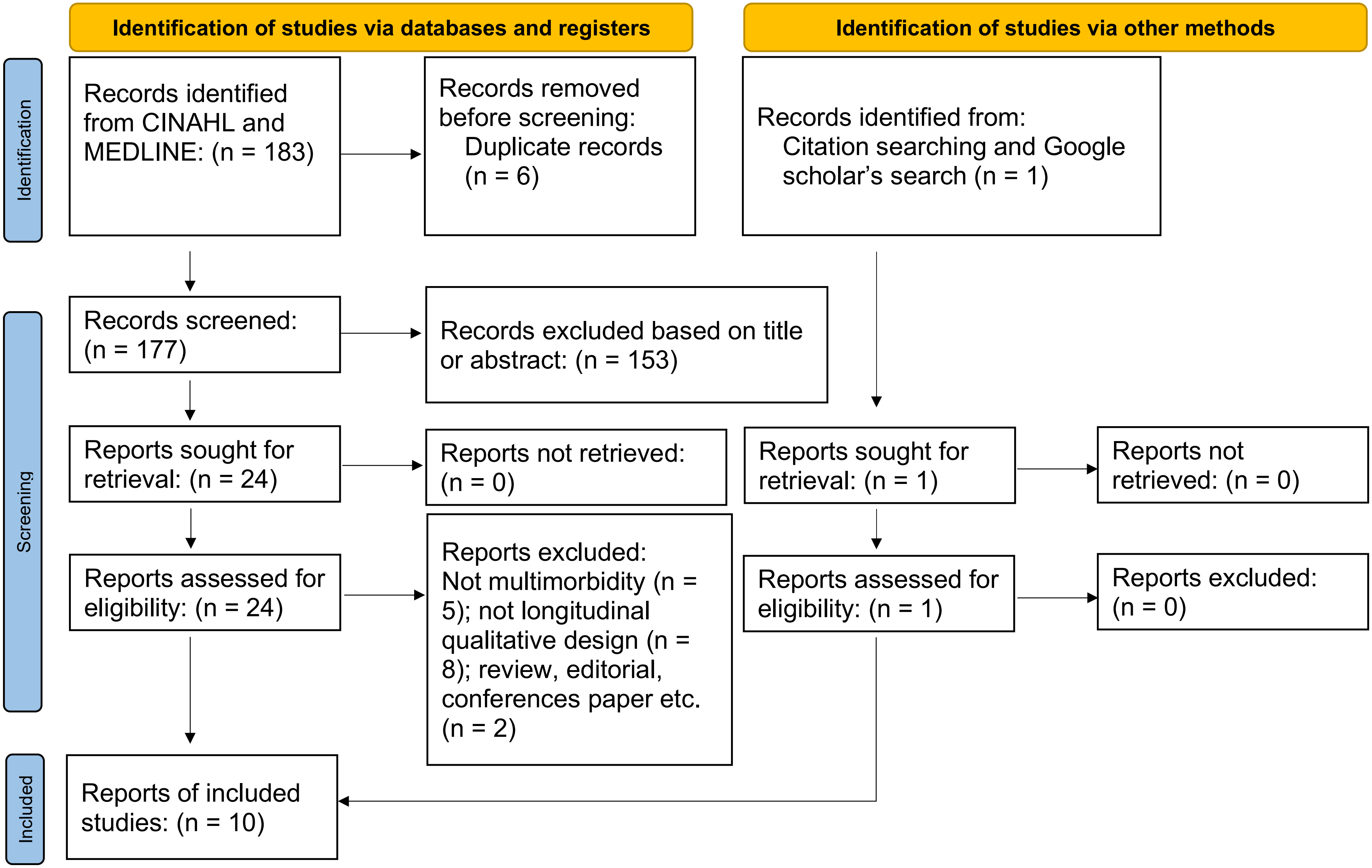

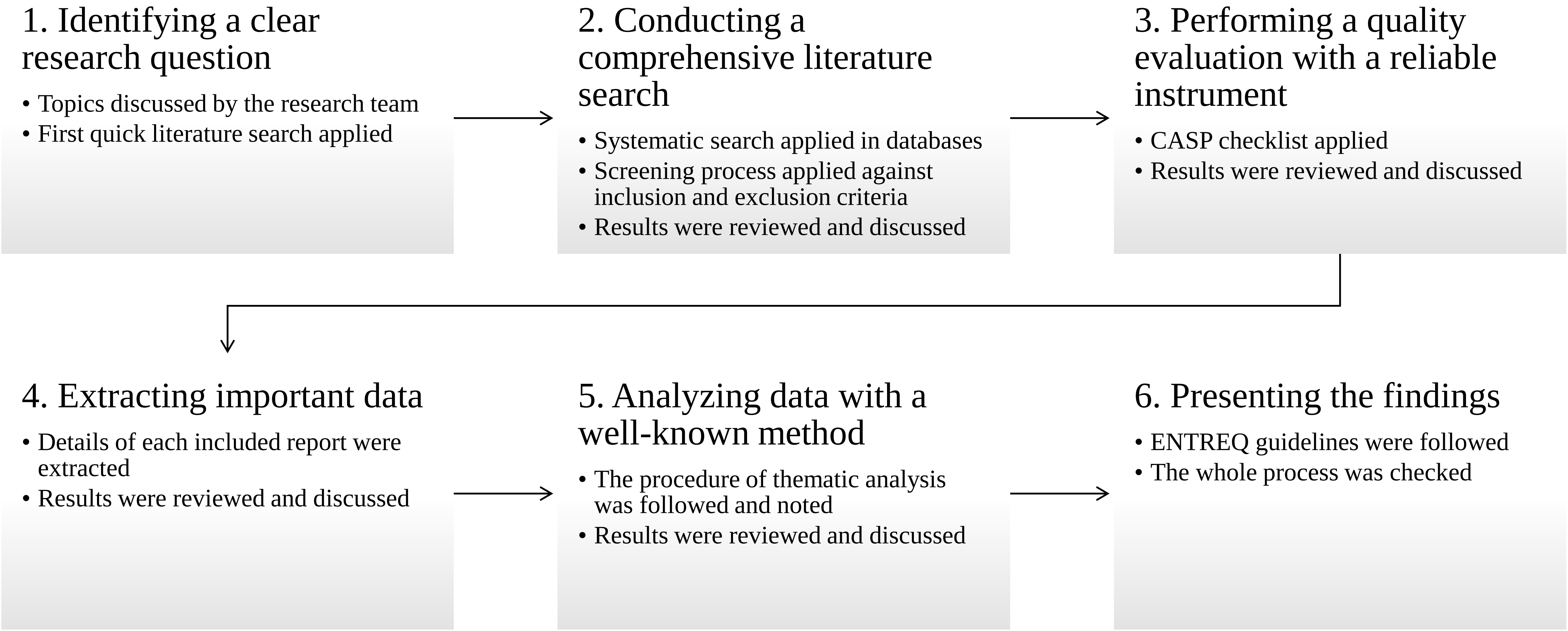

Figure S1. Meta-synthesis procedure

Living experiences with multimorbidity through time

A plain summary

Participants discussed the physical and psychological impacts of multimorbidity, their daily self-management, and the encounter with healthcare providers, emphasizing the importance of family support.

Theme	Codes
Perceiving multimorbidity	Identity and illness
	Impact of chronic illness
	Prioritization of health problems
	Medication burden
	Aging and health
Managing chronic conditions	Adaptation and resilience to illness
	Self-sufficiency and autonomy
	Goal setting and determination
Emotional struggles in everyday life with multimorbidity	Despair and hopelessness
	Frustration and stress
	Grief and loss
	Self-blame
	Lack of energy and motivation
	Fear and uncertainty
Interactions with the healthcare system and healthcare professionals	Trust and honesty
	Personalized care
	Confidence in treatment
	Continuity of care
	Empathy and understanding
	Health communication
	Healthcare system
Family Support	Family involvement and support
	Economic stability
	Advance care planning

Figure S2. Coding tree for thematic analysis

Note: Illustrative quotations are presented in the text.
