# Supplementary material for: Living with Multimorbidity through Time: A Meta-Synthesis of Qualitative Longitudinal Evidence
Source: Healthcare (Basel). 2024 Feb 9;12(4):446. doi: 10.3390/healthcare12040446 (PMC10887575; doi:10.3390/healthcare12040446)
Supplement: Supplementary file 1 [file healthcare-12-00446-s001.zip › Supplementary Tables.pdf]

Table S1. Reporting of database searching.

Reporting of database searching			
Database of searching	CINAHL	MEDLINE	Google Scholar
Time of searching	Oct 5, 2023		Oct 7, 2023
Boolean/Phrase[1]	(multimorbidity or multiple comorbidity or multiple chronic conditions, disease) AND (qualitative research or qualitative study or qualitative methods or interview) AND (longitudinal studies or longitudinal research or longitudinal method or longitudinal)	1 “chronic disease” [MeSH] OR multimorbidity [Text Word] OR “multiple comorbidity” [Text Word] OR “multiple chronic condition*” [Text Word] OR “multiple chronic disease*” [Text Word]<br>2 “qualitative research” [MeSH] OR “qualitative research” [Text Word] OR “qualitative study” [Text Word] OR “qualitative method*” [Text Word] OR “interview*” [Text Word]<br>3 “longitudinal studies” [MeSH] OR or “longitudinal research” [Text Word] OR “longitudinal method” [Text Word] OR “longitudinal” [Text Word]<br>4 1 AND 2 AND 3	multimorbidity (multiple comorbidity OR multiple chronic conditions) AND qualitative research (qualitative study OR qualitative methods) AND longitudinal research (longitudinal studies OR longitudinal method)

[1] No timeframe of publication applied.

Table S2. Inclusion and exclusion criteria**.**

Criteria	Inclusion	Exclusion
Participants	Adults (at least 18 years of age) with multimorbidity [1]	Adults without multimorbidity [2]
Setting	No limits applied	
Design	Primary studies with longitudinal qualitative research [3] (LQR) design or an LQR component;<br>Primary studies that serially collected data were also considered.	Primary studies with a quantitative design such as correlational, cohort, RCTs, etc.;<br>All types of reviews, commentaries, editorials, conference papers, etc.<br>Grey literature such as policy reports, working papers, newsletters, government reports, etc.
Phenomenon of interests	Experiences, attitudes, views, opinions, beliefs, or perspectives of people with multimorbidity [4].	Experiences, attitudes, views, opinions, beliefs, or perspectives regarding multimorbidity from healthcare professionals such as physicians, nurses, and pharmacists and inform carers, solely.
Language	English [5]	Other than English

[1] Multimorbidity refers to the presence of two or more long-term, chronic diseases, which include physical and mental health conditions such as chronic obstructive pulmonary disease or schizophrenia.

[2] People without multimorbidity refers to those having either no chronic disease or only one disease.

[3] Qualitative longitudinal research refers to qualitative studies that involve repeated data collection over time, with an emphasis on the temporal aspects of a particular phenomenon.

[4] Studies investigating experiences of people with multimorbidity as part of a broader group (e.g. physicians, nurses, pharmacists, social workers, etc.) were considered to be included if the first-person contributions of people with multimorbidity could be independently identified.

[5] The text is available and accessible in English, or via translatable text if not written in English.

Table S3. Methodological quality assessment.

Publications	1	2	3	4	5	6	7	8	9	10a	10b	10c
Morris et al. (2011)	Y	Y	Y	Y	Y	N	Y	Y	Y	Y	Y	N
Mason et al. (2016)	Y	Y	Y	Y	Y	N	Y	Y	Y	Y	Y	Y
Naik et al. (2016)	Y	Y	Y	Y	Y	N	Y	Y	Y	Y	Y	N
Hays et al. (2017)	Y	Y	Y	Y	Y	Y	Y	Y	Y	Y	Y	Y
Daker-White et al. (2018)	Y	Y	Y	Y	Y	N	Y	Y	Y	Y	Y	N
Francis et al. (2020)	Y	Y	Y	Y	Y	Y	Y	Y	Y	Y	Y	N
Porter et al. (2020)	Y	Y	Y	Y	Y	N	Y	Y	Y	Y	N	N
Brandberg et al. (2021)	Y	Y	Y	Y	Y	N	Y	Y	Y	Y	N	N
Bravo et al. (2022)	Y	Y	Y	Y	Y	N	Y	Y	Y	Y	Y	Y
Collier et al. (2023)	Y	Y	Y	Y	Y	N	Y	Y	Y	Y	Y	N

Note: The included publications were listed chronologically.
Abbreviations: Y = Yes, N = No, U = Unclear.

CASP qualitative study checklist

1. Was there a clear statement of the aims of the research?
2. Is a qualitative methodology appropriate?
3. Was the research design appropriate to address the aims of the research?
4. Was the recruitment strategy appropriate to the aims of the research?
5. Was the data collected in a way that addressed the research issue?
6. Has the relationship between researcher and participants been adequately considered?
7. Have ethical issues been taken into consideration?
8. Was the data analysis sufficiently rigorous?
9. Is there a clear statement of findings?
10. How valuable is the research?

The value of the corresponding research was examined using three sub-questions:

a. Does author link the findings to current practice, policy, or literature?
b. Does author identify new research areas?
c. Can the findings be transferred to other population?
